# Factors Affecting Rural Patients' Primary Compliance with e-Prescription: A Developing Country Perspective

**DOI:** 10.1089/tmj.2018.0081

**Published:** 2019-05-14

**Authors:** Nazmul Hossain, Masuda Begum Sampa, Fumihiko Yokota, Akira Fukuda, Ashir Ahmed

**Affiliations:** ^1^ Department of Advanced Information Technology, Kyushu University, Fukuoka, Japan.; ^2^ Department of Marketing, University of Dhaka, Dhaka, Bangladesh.; ^3^ Institute of Decision Science for Sustainable Society (IDS3), Kyushu University, Fukuoka, Japan.

**Keywords:** *e-prescription*, *primary compliance*, *rural patients*, *Bangladesh*

## Abstract

***Background:*** *The electronic prescription system has emerged to reduce the ambiguity and misunderstanding associated with handwritten prescriptions. The opportunities and challenges of e-prescription system, its impact on reducing medication error, and improving patient's safety have been widely studied. However, not enough studies were conducted to explore and quantify the factors that affect rural patients' compliance with e-prescription, especially from the perspective of Asian developing countries where most of the world's population resides.*

***Objective:*** *The objective of this study is to explore and assess the factors that affect rural patients' primary compliance with e-prescription in Bangladesh.*

***Methods:*** *Data were collected from 95 randomly selected rural patients who received e-prescription through a field survey with a structured questionnaire from Bheramara subdistrict, Bangladesh, during June and July 2016. Logistic regression analysis was performed to test the research hypotheses.*

***Results:*** *The study found patients' gender as the most significantly influential factor (regression coefficient [Coef.] = 2.02, odds ratio [OR] = 7.51,* p *< 0.05) followed by visiting frequency (Coef. = 0.99, OR = 2.70,* p *< 0.05); education (Coef. = 0.92, OR = 2.51,* p *< 0.05); and distance to healthcare facility (Coef. = 0.82, OR = 2.26,* p *< 0.01). However, patients' age, monthly family expenditure, and use of cell phone were found insignificant. The model explains 59.40% deviance (*R*^2^ = 0.5940) in the response variable with its constructs. And the “Hosmer–Lemeshow” goodness-of-fit score (0.99) is also above the standard threshold (0.05), which indicates the data fit well with the model.*

***Conclusions:*** *The findings of this study are expected to be helpful for e-health service providers to gain a better understanding of the factors that influence their patients to comply with e-prescriptions.*

## Introduction

Patients' noncompliance with prescription is a multifaceted healthcare problem. The reasons may be associated with the patient, treatment, and/or healthcare provider. However, as a result, patients are facing undesirable clinical outcomes and are deprived of optimal health recovery, which, in turn, lead to increased morbidity as well as increased financial and societal costs.^1^ In healthcare, the phrase “compliance with prescription” has a broader dimension. Vrijens et al. defined compliance as the degree to which a patient is able to follow the guidelines of prescribed treatment.^2^ Patients may be noncompliant in any phase of their treatment. They may decide not to collect their medicines from the pharmacy and not to start their treatment at all, which is considered as primary noncompliance. They may take more or less medication than was prescribed or use their medication at a wrong time. They may also suspend or even terminate their treatment ahead of prescribed time.^3,4^ This study, however, focuses on the factors that affect rural patients' primary noncompliance with e-prescription issued by a human-assisted remote healthcare system, namely Portable Health Clinic (PHC) in Bangladesh.

### About PHC

PHC is an e-health initiative, jointly developed by Kyushu University, Japan, and Grameen Communications, Bangladesh, to provide affordable healthcare solutions to low-income and low-literate people living in remote and underserved communities in Bangladesh by using information and communication technologies.^5,6^

**Fig. 1.**
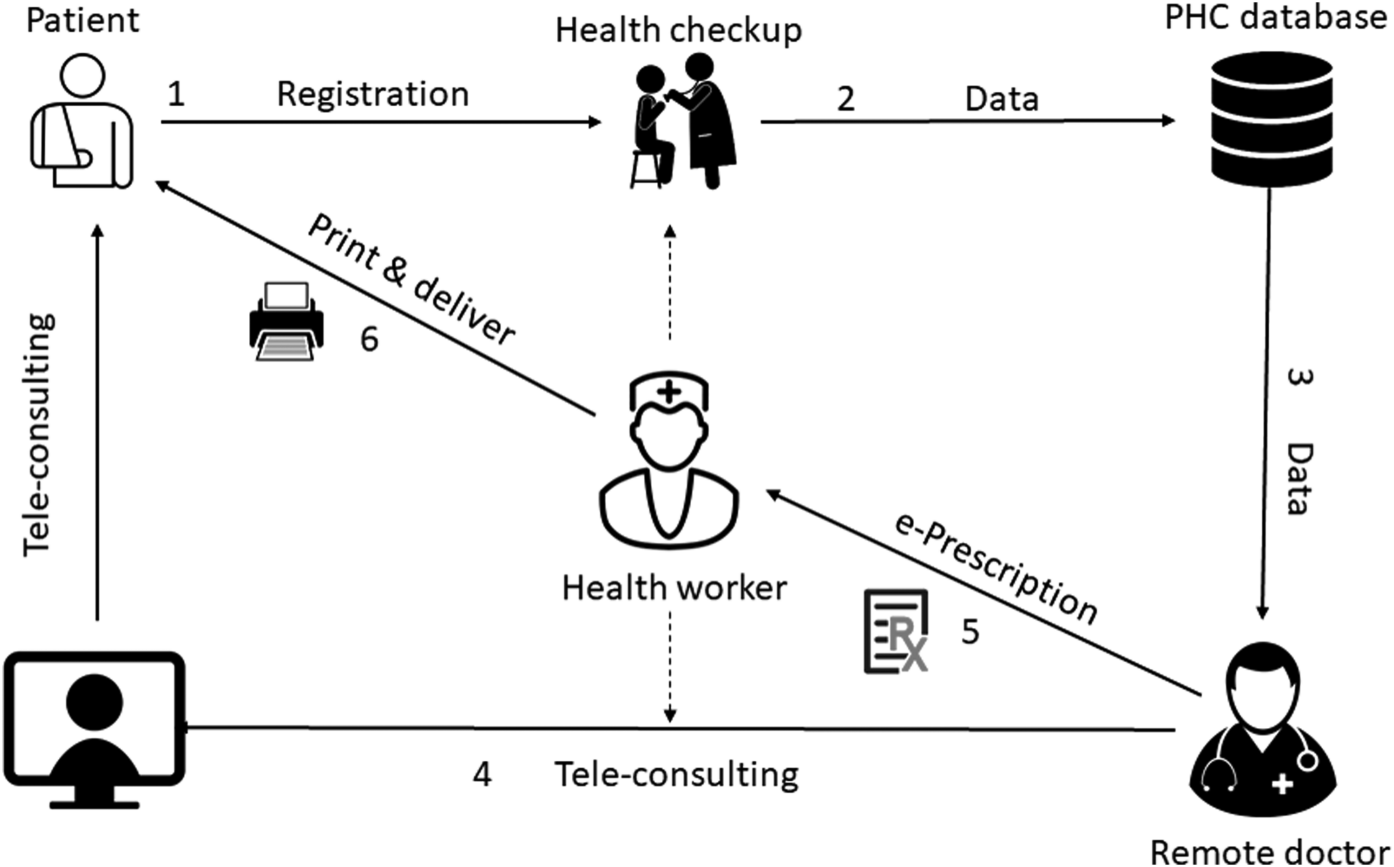
Healthcare service delivery flowchart of PHC. PHC, Portable Health Clinic.

To get healthcare services from PHC, patients first have to register their vital information such as name, age, sex, location, and disease complaints with the PHC system that generates a unique patient ID. Second, health checkup is conducted with an assistance of a healthcare worker and checkup data are automatically sent and stored to the central PHC server. The next step is teleconsultancy (voice and video) between the patients in need and the remote doctor located in the headquarters of PHC. After having conversation with patients and analyzing their clinical data, if necessary the doctor might issue an e-prescription, a printed version of that e-prescription is finally handed to the patient. The overall healthcare service delivery process of PHC is shown in *Figure 1*.

PHC is designed and targeted to provide the basic healthcare services to the underserved rural communities in Bangladesh with a view to reducing morbidity by combating against noncommunicable diseases. Therefore, majority of its patients are coming with health issues such as hypertension, anemia, arrhythmia, lower back pain, knee joint pain, burning sensation, and diabetes. PHC has started its experimental service since 2010. Until January 31, 2018, it reached 32 remote locations in 9 districts and served 41,240 rural patients, among whom 55.2% were male and 44.8% were female.^7^ For our research, we selected Bheramara subdistrict of Kushtia as our data collection site, which is one of the mentioned nine districts located in the northwestern part of Bangladesh. PHC started serving in Bheramara from 2012 and has served 4,701 rural patients until the mentioned date.

### PHC E-Prescription

Handwritten prescriptions have been used as a primary means of communication between prescribers and pharmacists. Over time, the risks associated with handwritten prescriptions such as difficulties with legibility and risk of misinterpretation encouraged the adoption of electronic prescriptions.^8^ An e-prescribing system sends an accurate, error-free, and understandable digital prescription directly to the patients or partnered pharmacies. It reduces the likelihood of adverse drug effects caused due to errors and misunderstandings in handwritten prescriptions.^9^ In Bangladesh, most of the rural patients are familiar and habituated with conventional handwritten prescriptions, whereas PHC is providing e-prescriptions issued by a remote doctor by using an e-prescription system software. A comparison between conventional handwritten prescription and PHC's e-prescription is given in *Table 1*.

**Table 1.** Difference Between Handwritten and Portable Health Clinic e-Prescription

FEATURE	HANDWRITTEN PRESCRIPTION	PHC E-PRESCRIPTION
Electronic entry	×	✓
Address individual patient	✓	✓
Medication monitoring	×	×
Access to patient's history	×	✓
Connect to pharmacy	×	×
Integrate with electronic medical record	×	✓

PHC, Portable Health Clinic.

According to a report by the Systems for Improved Access to Pharmaceuticals and Services (SIAPS) 2015, Bangladesh has ∼103,451 licensed retail drugstores and an estimated approximately equal number of unlicensed stores are involved in selling drugs “over-the-counter.” A majority (68%) of the clients visiting the drug shops came by self-referral and without a prescription, whereas the rest came with a prescription. Dispensing drugs on the basis of a patient's request (83%) or a patient's symptoms of illness (59%) is quite common.^10^ However, as an experimental remote healthcare service provider, PHC has not yet incorporated partner pharmacies with its system, that is, prescriptions are not routed to the pharmacists, rather a printed version is handed over to the patient. It, thus, cannot monitor patients' medication progress.

## Background and Objective

The features, benefits, and challenges of the e-prescription system, its impact on reducing medication error, and improving patient's safety and overall care quality have been widely studied. Odukoya and Chui.^11^ explained how e-prescribing can enhance the safety of patients, physicians, and pharmacists. Jariwala et al.^12^ described the factors affecting the adoption of the e-prescribing system by primary care physicians and their experience with the system in the United States. Kaushal et al.^13^ found that e-prescriptions reduce a significant amount of prescribing errors in comparison with handwritten prescriptions. Fernando et al.^14^ studied how electronically delivered prescriptions reduced pharmacy waiting time and improved patient satisfaction. Lapane et al.^15^ measured the perception and readiness to accept electronic prescriptions among elderly geriatric patients in six states in the United States. Smith^16^ explored the barriers to accepting e-prescription among general patients in the Pittsburgh metropolitan area in the United States. Kierkegaard^9^ examined the prospects and problems concerning the cross-border use of e-prescription among 27 member countries of the European Union. Ateniese and de Medeiros^17^ studied the issues related to privacy of medical data in e-prescription. However, not enough studies were conducted to explore and quantify the factors that affect rural patients' compliance with e-prescription, especially from the perspective of Asian developing countries where most of the world's population resides. Therefore, the objective of this study is to explore and assess the factors that affect rural patients' primary compliance with e-prescription in a developing country like Bangladesh.

## Methods

**Fig. 2.**
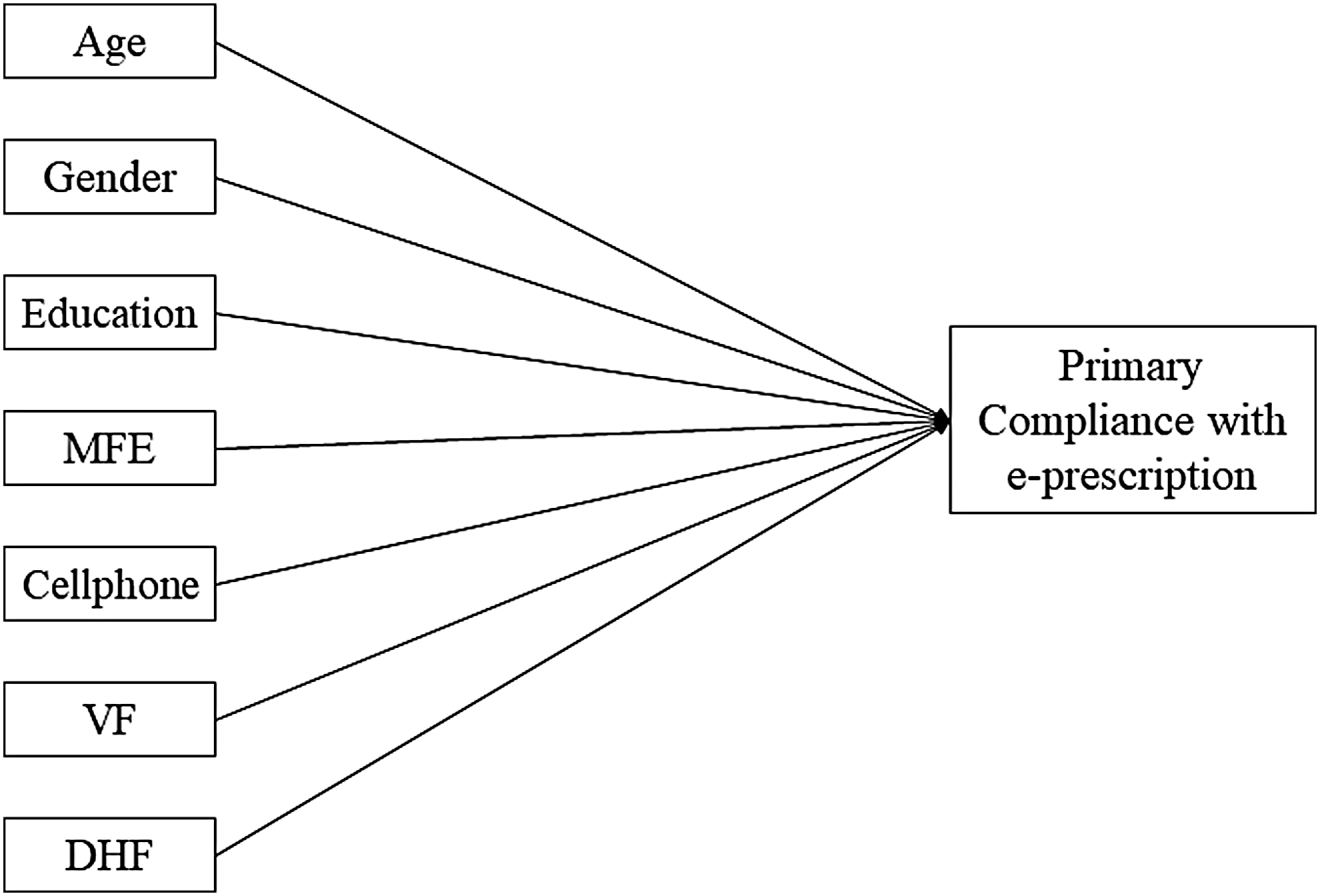
Research framework. DHF, distance to healthcare facility; MFE, monthly family expenditure; VF, visiting frequency.

To achieve the research objective, we have selected five sociodemographic factors, that is, age, gender, education, purchase power, and use of cell phone based on existing literature relating to patients' primary compliance with prescriptions. Several studies^2,18–22^ confirmed the profound impact of patients' sociodemography on their primary compliance with prescribed medication. Patel et al.^23^ found a positive correlation between drug adherence and physician visiting frequency, which motivated us to check whether there is any significant relationship between patients' visiting frequency of PHC and their primary compliance with the prescription. Syed et al.^24^ examined the relationship between medication compliance and distance to pharmacy and prescriber, which reinforced us to add one more variable to our research framework. However, in this study, we have employed a total of seven independent variables to measure their impacts and magnitudes on rural patients' primary compliance with e-prescription, which is shown as our research framework in *Figure 2*.

From the mentioned framework, keeping the research objective in mind, we have developed the following seven research hypotheses to be tested.

H1: Patients' age has a positive impact on primary compliance with e-prescription.

H2: Patients' gender significantly affects their compliance behavior.

H3: Level of education has a positive influence on patients' primary compliance with e-prescription.

H4: Patients' monthly family expenditure affects their primary compliance with e-prescription.

H5: Patients' use of cell phone has a significant impact on their compliance behavior.

H6: Visiting frequency has a positive impact on the patients' primary compliance with e-prescription.

H7: Distance to healthcare facilities has a significant impact on primary compliance with e-prescription.

To test the research hypotheses, data were collected through a field survey between June and July 2016 from Bheramara subdistrict of Kushtia, a northwestern district of Bangladesh. A structured questionnaire was developed initially in English, which later was translated into Bengali (the local language of Bangladesh). The survey questionnaire mostly covered the patients' sociodemographic information, their awareness, and usage of e-health, including usage frequency and finally their compliance behavior toward e-prescription. The questionnaire is given in Appendix 1. A pilot study was conducted on 7 randomly selected from >18 rural patients to test the understandability of the questionnaire. Their feedback was considered for reviewing the questionnaire. To maintain the right of privacy of the respondents, they have been briefed on the research purpose and were asked whether they want to participate in the survey and allow us to use their responses in our scientific publications.

**Fig. 3.**
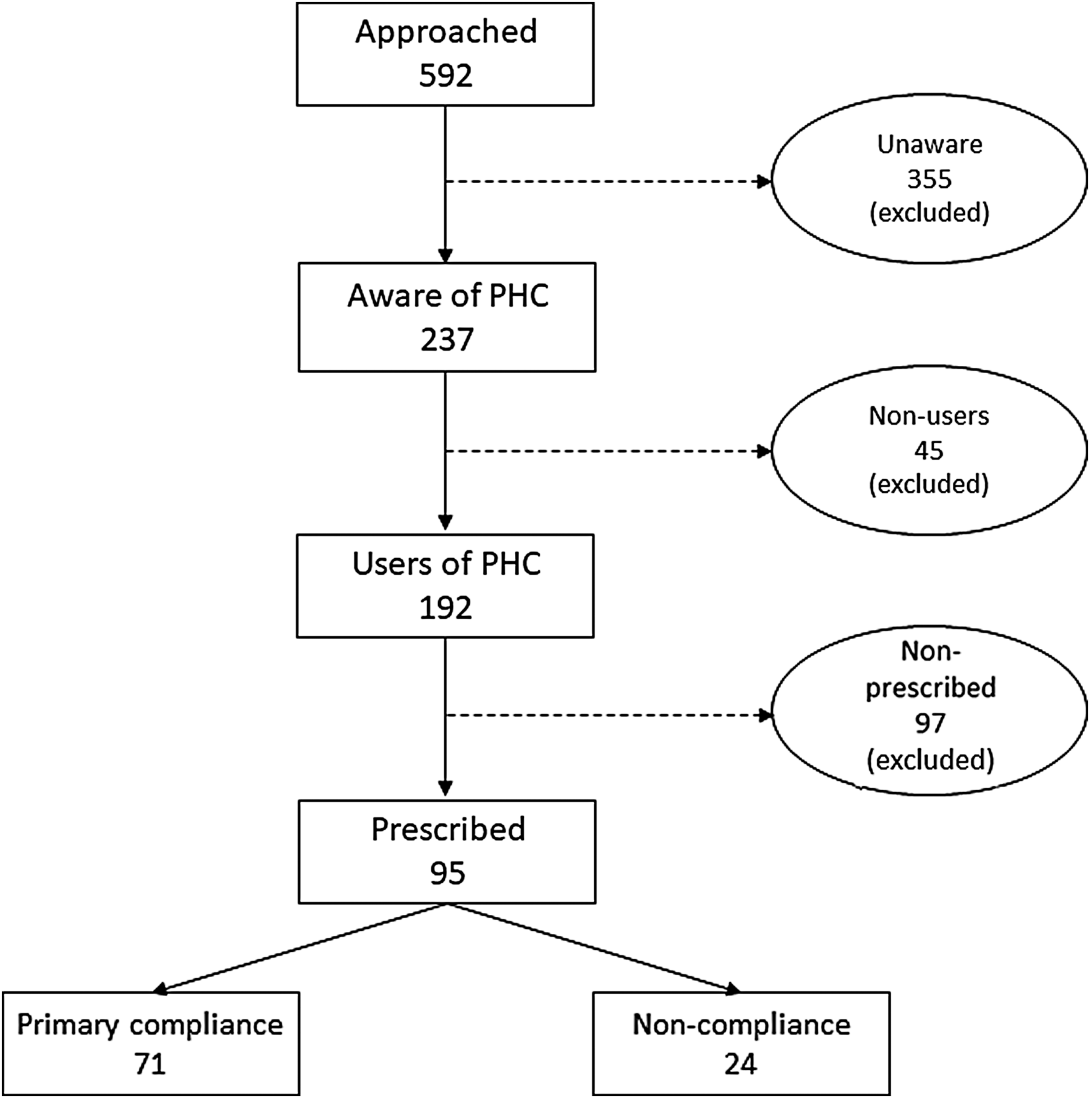
Steps in sample selection. PHC, Portable Health Clinic.

Since the dependent variable in this study is “compliance with e-prescription”’ and the response is categorized in either “Yes” or “No,” we are dealing with a binary classification problem. Several studies^25–27^ suggested that binary logistic regression fits better in this circumstance. Therefore, we also chose binary logistic regression model to test our research hypotheses. Beleites et al.^28^ suggested a minimum sample size of 75–100 to have a good but not perfect classifier model based on logistic regression. Figueroa et al.^29^ examined a total of 568 supervised learning-based classification models and found models with sample size between 80 and 560 achieved optimum performance. According to Peduzzi et al.^30^ and Kenny,^31^ in behavioral science with multivariate analysis, the sample size should be at least 10 times the number of items (independent variables) in the study. In our study, the model consists of 7 items and the effective sample size is 95, which is well supported by the studies already mentioned. To reach our targeted sample size, we randomly approached 592 rural respondents in our study area, among whom 355 were found unaware of PHC and were thus eliminated. Among the rest, 237 respondents who were aware of PHC, 45 found nonusers and were thus eliminated. Therefore, a total of 192 respondents were found who received healthcare services from PHC at least once. However, 95 (49%) patients out of 192 were reported to receive e-prescription from the remote doctor, thus, in this research, our effective sample size is 95. The sample selection process is shown in *Figure 3*.

## Results

### Respondents' Demography

Respondents' demographic characteristics are given in *Table 2*.

**Table 2.** Descriptive Statistics of Respondents (*n* = 95)

	FREQUENCY	PERCENTAGE
Gender		
Male	62	65.0
Female	33	35.0
Age group		
<30	19	20.0
30–45	43	45.5
46–60	26	27.5
>60	7	7.0
Education		
None	15	15.8
Primary	25	26.3
Secondary	38	40.0
College and higher	17	17.9
Monthly family expenditure (in BDT)		
<10,000	43	45.3
10,001–15,000	40	42.1
>15,000	12	12.6
Use of cell phone		
No phone	16	16.8
Feature phone	65	68.4
Smart cell phone	14	14.8
Compliance with e-prescription		
Yes	71	74.7
No	24	25.3

BDT, Bangladeshi taka (the local currency of Bangladesh).

The table shows the distribution of dependent variable, that is, patients' compliance with e-prescription, 74.7% were found compliant who reported collecting all the prescribed medicines, whereas 25.3% were found noncompliant. The table also shows the descriptive statistics of five independent variables, that is, age, gender, education, family expenditure, and use of cell phone. However, we have two more independent variables in this model, that is, PHC visiting frequency and distance to healthcare facility. The median visiting frequency per patient is 2 with a range of 1–10. The mean distance from patient's place to the nearest conventional healthcare facility is 3.3 km, with a standard deviation of 2.3 km.

### Correlation Among Independent Variables

Pearson correlation analysis is conducted to test whether any multicollinearity exists among independent variables before moving them to the final model, which is given in *Table 3*.

**Table 3.** Correlation Matrix of Independent Variables

	AGE	GENDER	EDU	MFE	CELLPHONE	PVF	DHF
Age	1						
Gender	0.32	1					
Education (Edu)	−0.18	0.29	1				
MFE	0.09	0.32	0.33	1			
Use of cell phone (CellPh)	−0.39	0.16	0.35	0.22	1		
PVF	−0.03	0.31	0.14	0.27	0.19	1	
DHF	−0.09	0.18	0.24	0.16	0.14	0.23	1

DHF, distance to healthcare facility; MFE, monthly family expenditure; PVF, PHC visiting frequency.

The matrix shows that no multicollinearity exists among independent variables since all the correlation coefficients are <0.40, which was referred as a threshold value by many researchers.^32^

### Results of Hypotheses Testing

A logistic regression modeling is used to test the hypotheses. A significance level of 0.05 is considered for this model. Decisions regarding hypotheses testing have been made by comparing the variables' *p*-value with models' significance level. Regression coefficient indicates the nature of the relationship between independent and dependent variable, whereas odds ratio explains the magnitude of the effect of independent variables on the dependent variable. The results of hypotheses testing are given in *Table 4*.

**Table 4.** Results of Hypotheses Testing Through Logistic Regression

HYPOTHESES	VARIABLE	COEF.	OR	95% CI	*p*	RESULT
1	Age	−0.390	0.6769	0.2219–2.0651	0.486	Not supported
2	Gender (male)	2.017	7.5134	1.0773–52.3988	0.032	Supported
3	Education	0.921	2.5120	0.9648–6.5399	0.041	Supported
4	MFE	1.106	3.0225	0.6165–14.8196	0.152	Not supported
5	Use of cell phone	0.334	1.3971	0.2784–7.0109	0.685	Not supported
6	PVF	0.994	2.7024	0.8340–8.7559	0.042	Supported
7	Distance to healthcare facility	0.815	2.2595	1.1300–4.5183	0.006	Supported

CI, confidence interval; Coef., regression coefficient; OR, odds ratio.

The finding says patients' gender, level of education, PHC visiting frequency, and distance to healthcare facility have significant influences on their primary compliance with e-prescription, whereas age, monthly family expenditure, and use of cell phone were found insignificant. Men are 7.5 times more likely to comply with e-prescription than women. Education has a positive correlation with compliance; higher educated patients are 2.5 times more likely to comply. Visiting frequency also has a positive impact, every one additional visit to PHC increases the patients' compliance likelihood by 2.7 times. Finally, distance matters, every 1 km of additional distance between patients' house and the conventional healthcare facility increases the likelihood of e-prescription compliance by 2.2 times.

### Model Summary and Goodness-Of-Fit

Our model has a deviance *R*^2^ of 0.594, which means the model explains 59.4% of the deviance in the response variable. For binary logistic regression, the “Hosmer–Lemeshow” test is a more trustworthy indicator of how well the model fits the data.^33^ In this model, the goodness-of-fit score is 0.99 that is greater than the significance level of 0.05, which indicates that there is not enough evidence to conclude that the model does not fit the data.

## Discussion and Limitations

A recent study conducted by Raebel et al. on 12,061 hypertension, diabetes, and lipid-disordered patients found that e-prescription reduced the primary noncompliance rate from 22% to 13% in comparison with handwritten prescriptions.^34^ Fernando et al. found 12.5% primary noncompliance with e-prescription among 224 emergency department patients.^14^ However, in this study, we found 25.3% primary noncompliance with e-prescription. This discrepancy exists since partner pharmacies have not yet been incorporated into the PHC system. According to a report by the Boston Consulting Group, the electronic transmission of a prescription to a pharmacy increases the possibility of picking up by the patient. It reduces the patient's obligation of providing the prescription to the pharmacy, a problem cited by more than one-third of patients who either forgot to drop it off or had difficulty doing so.^35^ This study found male patients to be more compliant with prescription, which is consistent with some previous studies,^22,36–38^ whereas some studies suggested otherwise.^39,40^ This difference in terms of prescription compliance by gender in rural Bangladesh exists because most of the rural female are unemployed house makers who have less mobility and more financial dependency on their male counterparts.^41^ Several studies^42–45^ found patients with higher educational level have higher propensity to comply with their prescriptions, which resembles our finding too. Innately, it is expected that patients with higher educational level should have better understanding and knowledge about their health, disease, and treatment, and, therefore, be more compliant.^19^

The outcomes of the study were based on patients' self-reporting through a questionnaire survey that might have some response bias. Moreover, the time gap between being prescribed and answering the questionnaire may have allowed for recall bias. The study was conducted on a particular geography, thus, concerns may arise about generalization. Further research, therefore, can be carried out by covering a broader geography and adding a few additional independent variables such as patient–prescriber relationship, patients' trust and attitude toward the system, and distance between patients' house and drugstores to have more comprehensive insights.

## Conclusions

Primary compliance with prescription, in healthcare, is a vital issue since noncompliance causes unexpected delay in health recovery along with financial and social burdens on patients. The study found patients' gender, education, visiting frequency to care provider, and distance to healthcare facilities are strongly associated with their compliance behavior, whereas their age, monthly family expenditure, and use of cell phone were found insignificant. The findings of this study are expected to be helpful for e-health service providers to gain a better understanding of the factors that influence their patients to comply with e-prescriptions.

## Appendix 1

### Survey Questionnaire: For Exploring the Factors Affecting Primary Compliance with e-Prescription

1. Respondent's age: []
2. Respondent's gender:
  (a) Male
  (b) Female
3. Level of education:
  (a) None
  (b) Primary
  (c) Secondary
  (d) College and higher
4. Monthly family expenditure (in BDT):
  (a) Less than 10,000
  (b) 10,001–15,000
  (c) More than 15000
5. Do you have a mobile phone?
  (a) Yes
  (b) No
  (If the answer is “Yes”)
  5.1. What kind of phone you have?
  (a) Feature phone
  (b) Smartphone
  (c) Both
6. What is the distance between your house and the nearest traditional healthcare center such as hospital or clinic?
Ans.: …………… km.
7. Do you think ICT (mobile phone, laptop computer, internet network) can be used to obtain healthcare services (health checkup, consulting with doctors, obtaining prescription, etc.)?
  (a) Yes
  (b) No
8. Have you ever seen any promotional campaign (poster, leaflet, announcement, etc.) of Portable Health Clinic (PHC) in your area?
  (a) Yes
  (b) No
9. Have you ever received any healthcare service from PHC?
  (a) No
  (b) Yes
  (If the answer is “Yes”)
  9.1. How many times have you visited PHC?
  (a) Only once
  (b) More than once
  (c) Mention whether you remember (……….) times
10. Have you ever received any e-prescription from PHC?
  (a) No
  (b) Yes
  (If the answer is “Yes”)
  10.1. What did you do after getting that prescription?
  (a) I bought all the drugs according to the prescription
  (b) I bought some of the drugs but not all
  (c) I didn't buy any drug and waited for natural recovery
